# Systemic Inflammatory and Microcirculatory Determinants of Long-Term Outcomes in Neovascular Glaucoma: A Multicenter Cohort Study

**DOI:** 10.22336/rjo.2026.39

**Published:** 2026

**Authors:** Olga Volodymyrivna Guzun, Oleg Serhiyovich Zadorozhnyy, Camelia Margareta Bogdanici, Volodymyr Viktorovych Vychuzhanin, Natalia Ivanivna Khramenko, Liudmyla Mykolayivna Velichko, Andrii Rostyslavovich Korol, Valeriu Nicon Cușnir, Lilia Gheorghe Dumbrăveanu

**Affiliations:** 1 “The Filatov Institute of Eye Diseases and Tissue Therapy of the National Academy of Medical Sciences of Ukraine” State Institution, Odesa, Ukraine; 2 Department of Ophthalmology, “Grigore T. Popa” University of Medicine and Pharmacy, Iaşi, Romania; 3 “Odesa Polytechnic” National University, Odesa, Ukraine; 4 Department of Ophthalmology and Clinical Optometry, “Nicolae Testemițanu” State University of Medicine and Pharmacy, Chişinău, Republic of Moldova

**Keywords:** neovascular glaucoma, anterior chamber angle, cyclophotocoagulation, glaucoma surgery, systemic inflammation, microcirculation, treatment prognosis, NVG = neovascular glaucoma, IOP = intraocular pressure, BCVA = best-corrected visual acuity, LogMAR = logarithm of the minimum angle of resolution, TSCPC = transscleral cyclophotocoagulation, CPC = cyclophotocoagulation, RQ = rheographic coefficient, SII = systemic immune-inflammation index, SIRI = systemic inflammation response index, AISI = aggregate index of systemic inflammation, HbA1c = glycated hemoglobin, CVD = cardiovascular disease, OR = odds ratio, CI = confidence interval, ROC = receiver operating characteristic, AUC = area under the curve

## Abstract

**Background:**

Neovascular glaucoma (NVG) represents one of the most severe forms of secondary glaucoma and is characterized by an aggressive clinical course, a high risk of irreversible vision loss, and substantial variability in treatment outcomes. At the same time, the roles of systemic inflammation, ocular microcirculation, and neovascularization of the iridocorneal angle (according to the Weiss D. classification, 1978) in determining the long-term prognosis remain insufficiently investigated.

**Purpose:**

To identify clinical, microcirculatory, and systemic inflammatory predictors of long-term treatment effectiveness in secondary NVG and to compare the outcomes of surgical interventions and diode transscleral cyclophotocoagulation depending on the stage of iridocorneal angle neovascularization.

**Methods:**

A multicenter retrospective cohort study included 87 eyes with ischemic NVG secondary to proliferative diabetic retinopathy or retinal vein occlusion treated between 2018 and 2024 in Ukraine and Moldova. Patients were divided into a surgical group (n=39) and a diode transscleral cyclophotocoagulation group (n=48). Clinical parameters, systemic inflammatory indices (SII, SIRI, AISI), HbA1c, cardiovascular comorbidity, and the rheographic coefficient (RQ), a functional microcirculation marker, were analyzed. Treatment success was defined as intraocular pressure control with stabilization of visual acuity, absence of rubeosis progression, and no need for additional invasive surgery. Multivariable logistic regression and ROC analysis were performed.

**Results:**

Treatment effectiveness was strongly associated with the stage of iridocorneal angle neovascularization. In the early stages, both approaches showed high success rates, whereas in the advanced stages, surgical outcomes declined markedly. In multivariable analysis, surgery increased the odds of success more than fivefold (OR=5.4; p=0.003), whereas advanced angle stages reduced it nearly sixfold (OR=0.18; p=0.007). Higher systemic inflammation was associated with poorer prognosis (SII OR=0.76; p=0.009), whereas better microcirculation was associated with improved outcomes (RQ OR=1.42; p=0.023). The combined predictive model demonstrated good discrimination (AUC = 0.84; 95% CI, 0.74–0.92).

**Discussion:**

In the present study, long-term outcomes of neovascular glaucoma were shown to be determined by the complex interaction between the stage of iridocorneal angle neovascularization, systemic inflammation, and ocular microcirculation. Our findings indicated that the Weiss stage is a key morphological factor that modifies treatment effectiveness. In contrast, systemic inflammatory indices (SII, SIRI, AISI) reflect the underlying biological burden of disease and are associated with poorer prognosis. In contrast, higher values of the microcirculatory parameter (RQ) were significantly associated with more favorable treatment outcomes, likely reflecting preserved perfusion capacity and a lower degree of ischemic tissue damage. This observation is consistent with the concept of ischemia-driven progression in NVG. Importantly, integrating these parameters into a multivariable model demonstrated good discriminative performance (AUC = 0.84), supporting the value of a combined clinical-biological approach to risk stratification. Taken together, these results suggest that NVG should be considered not only a localized ocular disorder but also a systemically influenced condition in which the interplay among angiogenesis, inflammation, and microcirculatory dysfunction determines individual treatment response.

**Conclusions:**

Long-term outcomes in neovascular glaucoma appear to be determined by the interaction between the stage of iridocorneal angle neovascularization, systemic inflammation, and ocular microcirculation. Surgical treatment provides the greatest benefit in early disease, whereas cyclophotocoagulation remains a stable option in advanced stages. Combined clinical-biological modeling may support personalized treatment selection in NVG.

## Introduction

Neovascular glaucoma (NVG) is one of the most severe forms of secondary glaucoma and is associated with a high risk of irreversible vision loss [1]. Most commonly, neovascular glaucoma develops as a complication of proliferative diabetic retinopathy (PDR), retinal vein occlusions (RVO), and other ischemic retinopathies. These conditions are characterized by chronic retinal hypoxia and increased expression of vascular endothelial growth factor (VEGF), which plays a central role in the development of ocular neovascularization [2]. In response to ischemic stimulation, an angiogenic-inflammatory microenvironment is formed, characterized by elevated levels of pro-inflammatory cytokines and pro-angiogenic factors that sustain the pathological neovascular process [3].

Excessive VEGF-dependent neovascularization leads to iris rubeosis, formation of fibrovascular membranes, and progressive obliteration of the iridocorneal angle, followed by elevation of intraocular pressure (IOP) [4].

Despite significant advances in anti-VEGF therapy and retinal laser photocoagulation, IOP control in neovascular glaucoma often remains insufficient, particularly in cases with established anterior synechiae [5]. In such clinical situations, the main treatment options include filtration surgery [6], implantation of drainage devices [5], and cyclodestructive procedures [7,8], which aim to stabilize IOP and are typically employed when medical and laser therapies are ineffective [9].

Transscleral diode cyclophotocoagulation reduces aqueous humor production and provides effective intraocular pressure (IOP) control in advanced stages of neovascular glaucoma, including when used in combination with anti-VEGF therapy and other interventions [8,10].

However, the long-term efficacy of surgical treatment in NVG remains variable, largely due to the pronounced inflammatory-ischemic background of the disease and the tendency of anterior segment tissues toward fibrotic remodeling [5].

Moreover, the clinical course of NVG is highly heterogeneous, leading to substantial differences in treatment response [11].

Most available clinical studies focus on short-term outcomes of NVG treatment, while data on the long-term course of the disease and the durability of treatment effects remain limited [5]. At the same time, long-term follow-up allows a comprehensive assessment not only of intraocular pressure control in patients with neovascular glaucoma, but also of long-term clinical outcomes, including changes in visual function and the frequency of repeated interventions over several years after treatment. Such observations reveal a gradual decline in the proportion of successful outcomes over time and highlight the influence of various prognostic factors on disease progression [12,13].

This study aimed to evaluate clinical, systemic inflammatory, and microcirculatory determinants of long-term treatment outcomes in ischemic neovascular glaucoma and to compare the effectiveness of glaucoma surgery and diode transscleral cyclophotocoagulation across different stages of iridocorneal angle involvement.

## Materials and methods

### Study design and patient selection

This study had a multicenter retrospective cohort design. All cases of neovascular glaucoma (NVG) managed in specialized ophthalmic centers in Odesa (Ukraine) and Chișinău (Republic of Moldova) between 2019 and 2023 were initially screened. After applying predefined inclusion and exclusion criteria, patients with ischemic NVG of confirmed etiology — proliferative diabetic retinopathy or retinal vein occlusion — and complete clinical follow-up data were finally included (Table 1).

**Table 1:** Patient Selection Flow Diagram

Selection Stage	Number of Patients	Description
Initially assessed	n = 432	All patients diagnosed with NVG treated at ophthalmology centers in Odesa and Chișinău between 2018 and 2024
Excluded	n = 128	Absence of confirmed ischemic etiology of NVG (PDR or RVO)
Excluded	n = 83	Incomplete clinical or laboratory data
Excluded	n = 134	Follow-up period less than 24 months
Final cohort	n = 87	Patients included in the statistical analysis
Surgery group	n = 39	Patients who underwent antiglaucoma surgical procedures
TSC CPC group	n = 48	Patients who underwent transscleral cyclophotocoagulation
Outcomes analyzed	n = 87	Evaluation of treatment effectiveness, functional outcomes, and predictors of success

*Notes*. The table presents the sequential formation of the study cohort from initial screening to final analysis. *Abbreviations*: NVG = neovascular glaucoma, TSC CPC = transscleral cyclophotocoagulation, PDR = proliferative diabetic retinopathy, RVO = retinal vein occlusion

Patients were divided into two treatment groups according to the primary intervention received: glaucoma surgery or diode transscleral cyclophotocoagulation (TSCPC). Surgical procedures included filtering operations and implantation of glaucoma drainage devices to create an alternative aqueous outflow pathway and stabilize intraocular pressure (IOP) [14]. The laser group underwent selective diode TSCPC, designed to reduce aqueous humor production through controlled ciliary body photocoagulation [15].

### Inclusion criteria

Eyes were included if they demonstrated:

- iris neovascularization and/or angle neovascularization,
- elevated intraocular pressure (> 21 mmHg),
- documented ischemic retinal disease (PDR or RVO),
- minimum follow-up of 24 months after intervention.

## Clinical assessment

All patients underwent standardized ophthalmic examination before treatment, including:

- best-corrected visual acuity (BCVA) converted to LogMAR,
- Goldmann applanation tonometry,
- slit-lamp biomicroscopy,
- gonioscopy with assessment of angle neovascularization and degree of synechial closure according to Weiss classification [16],
- fundus examination.

Angle involvement was categorized into:

- early stage (open or partially synechially closed angle; stages I–II),
- late stage (advanced fibrovascular angle closure; stages III–IV).

### Systemic and microcirculatory parameters

Systemic metabolic and inflammatory status were assessed using glycated hemoglobin (HbA1c) and systemic inflammatory indices (SII, SIRI, AISI). Cardiovascular comorbidities were recorded for each patient.

Ocular hemodynamics were evaluated using the rheographic coefficient (RQ) as a functional marker of ocular microcirculation [17].

### Study endpoints

Treatment effectiveness was defined as achieving controlled intraocular pressure without progression of iris rubeosis or iridocorneal angle neovascularization, and without the need for repeated invasive interventions in the case of surgical treatment. For cyclophotocoagulation, repeated laser treatment was considered acceptable if it resulted in stabilization of intraocular pressure and the eye’s clinical condition.

Secondary endpoints included changes in visual acuity, regression of anterior segment neovascularization, need for systemic hypotensive therapy, and incidence of complications.

### Statistical analysis

Data were analyzed using nonparametric methods and were presented as the median with the interquartile range (Q1-Q3). Continuous variables were compared using the Mann-Whitney U test, and categorical variables using Fisher’s exact test. Independent predictors of treatment success were assessed using multivariable logistic regression, with odds ratios (ORs) and 95% confidence intervals calculated. The optimal threshold was determined using the Youden index. Statistical significance was set at p < 0.05.

### Ethical considerations

The study was conducted in accordance with the Declaration of Helsinki and approved by the Bioethics Committee of the Filatov Institute of Eye Diseases and Tissue Therapy, National Academy of Medical Sciences of Ukraine. All patients provided informed consent for the use of clinical data for research purposes.

## Results

### Cohort characteristics

A total of 87 eyes with ischemic neovascular glaucoma (secondary to proliferative diabetic retinopathy or retinal vein occlusion) were finally included. Patients were assigned to either glaucoma surgery (n=39) or diode transscleral cyclophotocoagulation (TSCPC) (n=48). Baseline HbA1c and visual acuity did not differ significantly between groups; however, patients in the surgical group were younger and showed a distinct systemic and microcirculatory profile, including lower RQ and higher systemic inflammatory indices (SII, SIRI, AISI) (Table 2). These baseline differences reflect real-world treatment selection in routine clinical practice.

**Table 2:** Baseline demographic and clinical characteristics of patients according to treatment modality

Predictor (baseline)	Surgery (n=39)	TSC CPC (n=48)	p
	Median (Q1-Q3)		
Age, years	62 (57; 67)	66 (64; 71)	< 0.001
HbA1c, %	8.1 (7.6; 8.4)	7.6 (7.6; 7.7)	0.11
IOP, mm Hg	34 (33; 40)	32 (32; 39)	0.056
RQ, ‰	3.0 (2.05; 3.60)	3.7 (3.14; 4.20)	0.0003
SII (ratio)	441 (375; 673)	386 (354; 440)	0.001
SIRI (ratio)	0.76 (0.55; 1.14)	0.53 (0.49; 0.56)	< 0.001
AISI (ratio)	148 (100; 309)	95 (87; 101)	< 0.001
BCVA, (LogMAR)	1.70 (1.15; 2.00)	2.00 (1.70; 2.00)	0.18

Notes. Data are presented as median and interquartile range (Q1-Q3). Independent groups were compared using the Mann-Whitney U test. Abbreviations: IOP = intraocular pressure, RQ = rheographic coefficient (functional marker of ocular microcirculation), SII = systemic immune-inflammation index, SIRI = systemic inflammation response index, AISI = aggregate index of systemic inflammation, BCVA = best-corrected visual acuity, LogMAR = logarithm of the minimum angle of resolution, TSCPC = transscleral cyclophotocoagulation

### Treatment effectiveness according to the stage of iridocorneal angle neovascularization

Treatment effectiveness significantly depended on the stage of iridocorneal angle neovascularization. At early stages (stage II), both strategies demonstrated high success rates: 100% for surgery (9/9) and 92% for TSCPC (23/25). In contrast, at late stages (stages III-IV), the effectiveness of surgery decreased markedly to 33.3% (10/30), whereas TSCPC maintained a higher success rate of 65.2% (15/23) (Table 3; Fisher’s exact test for late-stage comparison, p=0.006). These findings indicate that structural angle damage acts as a key modifier of long-term treatment response in NVG.

**Table 3:** Treatment effectiveness according to the stage of iridocorneal angle involvement

Rubeosis stage	Treatment modality	Successful	Ineffective	Success rate
Early (stage II)	TSCPC	23	2	92%
Late (stages III-IV)	TSCPC	15	8	65.2%
Total TSCPC		38	10	79.2%
Early (stage II)	Surgery	9	0	100%
Late (stages III-IV)	Surgery	10	20	33.3%
Total Surgery		19	20	48.7%

*Notes*. Treatment success was defined as achieving the target intraocular pressure without progression of anterior segment neovascularization and without requiring additional invasive surgery during follow-up. For the TSCPC group, repeated laser treatment was considered acceptable if it resulted in stable IOP control. Differences between groups were assessed using Fisher’s exact test. *Abbreviations*: TSCPC = transscleral cyclophotocoagulation, IOP = intraocular pressure

### Factors associated with treatment effectiveness within each modality

Within the surgical group, successful outcomes were associated with younger age, lower baseline IOP, more favorable microcirculation (higher RQ), and significantly lower systemic inflammation. Cardiovascular comorbidity was more frequent among patients with ineffective surgical outcomes, supporting the relevance of systemic vascular status for long-term prognosis (Table 4).

**Table 4:** Clinical, systemic, and microcirculatory factors associated with treatment effectiveness within each modality

Predictor	Surgery treatment (n=39)		p	TSCPC (n=48)		p
	Successful (n=19)	Ineffective (n=20)		Successful (n=38)	Ineffective (n=10)	
Age, years	57 (56; 61)	67 (62; 68)	<0.001	67 (64.5; 71)*	64 (64; 66,8)	0.64
HbA1c, %	7.60 (7.50; 8.15)	8.25 (8.00; 8.47)	0.044	7.60 (7.60; 7.70)	8.20 (8.05; 8.32)	0.07
IOP V0	33 (30; 33.5)	40 (40; 42)	<0.001	32 (32; 39)	32 (32; 32)⁑	0.33
RQ V0	3.16 (3.00; 3.60)	2.60 (1.40; 3.67)	0.058	3.65 (3.24; 4.20)*	3.14 (3.14; 3.35) ⁑	0.23
SII (ratio)	380 (365; 417)	673 (672; 673)	<0.001	378 (353; 388)	634 (620; 646) ⁑	0.002
SIRI (ratio)	0.55 (0.49; 0.62)	1.14 (1.13; 1.14)	<0.001	0.53 (0.52; 0.56)	0.49 (0.48; 0.49) ⁑	0.03
AISI (ratio)	100 (90; 113)	309 (296; 335)	<0.001	94 (90; 101)	90 (87; 90) ⁑	0.06
BCVA V0 (LogMAR)	1.10 (0.82; 1.52)	1.70 (1.70; 2.30)	<0.001	2.00 (1.70; 2.00)	1.10 (1.10; 1.22) ⁑	0.25
CVD (%)	63%	90%	0.03	67%	100%	0.28

*Notes*. Continuous variables are presented as median and interquartile range (Q1-Q3). Between-group comparisons were performed using the Mann-Whitney U test, and categorical variables using Fisher’s exact test. Reported p-values refer to comparisons between effective and ineffective outcomes within each treatment modality; * indicates statistical significance for comparison of effective outcomes between treatment modalities; ⁑ indicates statistical significance for comparison of ineffective outcomes between treatment modalities. *Abbreviations*: IOP = intraocular pressure, RQ = rheographic coefficient, SII = systemic immune-inflammation index, SIRI = systemic inflammation response index, AISI = aggregate index of systemic inflammation, BCVA = best-corrected visual acuity, LogMAR = logarithm of the minimum angle of resolution, CVD = cardiovascular disease

Within the TSCPC group, the biggest differences between effective and ineffective outcomes were observed primarily in systemic inflammatory indices, whereas other baseline clinical parameters demonstrated less consistent associations (Table 4).

### Comparison of outcomes among eyes with effective treatment

Among eyes achieving treatment success, both modalities provided comparable final IOP control at 24 months (18.0 vs. 19.0 mmHg; p=0.92). However, the magnitude of IOP reduction differed: TSCPC produced a greater median decrease in IOP (ΔIOP 18.0 vs. 14.0 mmHg; p=0.001). Conversely, functional outcomes favored surgery: BCVA at 24 months was significantly better in the surgical group (LogMAR 0.77 vs. 1.40; p=0.002), while the change in BCVA over follow-up did not differ significantly (ΔLogMAR −0.33 vs. −0.30; p=0.62) (Table 5).

Regression of anterior segment rubeosis was frequent in both groups and did not differ significantly (64.1% vs. 70.8%; p=0.50). The overall complication rate was higher after surgery (30.8% vs. 14.6%; p=0.07), whereas the proportion of patients requiring hypotensive drops at 24 months did not differ significantly (25.6% vs. 37.5%; p=0.24) (Table 5).

**Table 5:** Comparison of outcomes among eyes with effective treatment: surgery vs. TSCPC

Predictor	Successful surgery	Successful CPC	p
IOP V24, mmHg	18.0 (17.5; 19.5)	19.0 (14.0; 20.0)	0.92
ΔIOP, mmHg	14.0 (12.0; 15.5)	18.0 (15.3; 19.0)	0.001
LogMAR V24	0.77 (0.52; 1.15)	1.40 (1.25; 1.55)	0.002
ΔLogMAR	−0.33 (−0.45; −0.20)	−0.30 (−0.35; −0.20)	0.62
Rubeosis regression, %	64.1%	70.8%	0.5
Complications, %	30.8%	14.6%	0.07
Acetazolamide use at 24 months, %	25.6%	37.5%	0.24

Notes. Data are presented as median and interquartile range (Q1-Q3) or proportion (%). Δ indicates the change in a parameter between baseline and the final visit. Comparisons were performed using the Mann-Whitney U test and Fisher’s exact test. Abbreviations: IOP = intraocular pressure, LogMAR = logarithm of the minimum angle of resolution, CPC = cyclophotocoagulation

### Multivariable predictors of long-term success

Multivariate logistic regression analysis showed that the type of intervention, the stage of iridocorneal angle neovascularization, systemic vascular status, the level of systemic inflammation, and microcirculatory parameters were independent predictors of long-term treatment success (Table 6).

**Table 6:** Multivariable logistic regression model of predictors of treatment success in neovascular glaucoma

Predictor	OR	95% CI	p
Surgery (vs. CPC)	5.4	1.8-16.2	0.003
Stage of iridocorneal angle neovascularization III–IV	0.18	0.05-0.62	0.007
Age (per 10-year increase)	0.63	0.44-0.90	0.012
HbA1c (per 1% increase)	0.78	0.54-1.12	0.18
Cardiovascular disease	0.41	0.17-0.98	0.045
IOP (per 5 mmHg increase)	0.89	0.70-1.14	0.36
RQ	1.42	1.05-1.92	0.023
SII (per 100 units of the index increase)	0.76	0.62-0.93	0.009

*Notes*. Results of a multivariable logistic regression analysis predicting treatment success in neovascular glaucoma are presented. Odds ratios (OR) are shown with 95% confidence intervals. *Abbreviations*: OR = odds ratio, CI = confidence interval, IOP = intraocular pressure, RQ = rheographic coefficient, SII = systemic immune-inflammation index, CPC = cyclophotocoagulation, CVD = cardiovascular disease

Surgery increased the odds of treatment success more than fivefold (OR=5.4; p=0.003), whereas an advanced stage of iridocorneal angle neovascularization (3-4) (III-IV) reduced the odds nearly sixfold (OR=0.18; p=0.007). Higher systemic inflammation was associated with poorer prognosis (SII per 100 units: OR=0.76; p=0.009), whereas better microcirculation (higher RQ) increased the likelihood of success (OR=1.42; p=0.023). Model calibration was acceptable (Nagelkerke R^2^=0.36), and the Hosmer–Lemeshow test did not indicate a significant lack of fit (p>0.05).

### ROC performance of the combined prognostic model

**Fig. 1:**
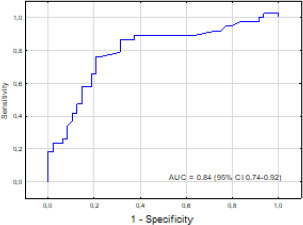
Receiver operating characteristic (ROC) curve for prediction of treatment outcome in neovascular glaucoma after cyclophotocoagulation. The model demonstrated good discriminative ability (AUC = 0.84; 95% CI 0.74-0.92). Optimal cut-off determined by the Youden index: sensitivity 79% and specificity 76% (J = 0.55)

The ROC curve was constructed using a multivariate logistic regression model that included treatment method, angle lesion stage, age, HbA1c, presence of cardiovascular diseases, intraocular pressure, rheographic coefficient (RQ), and systemic immune inflammation index (SII). The combined clinical-biological model demonstrated good discrimination for long-term treatment outcome, with an AUC of 0.84 (95% CI, 0.74-0.92) (Fig. 1).

These findings suggest that long-term outcomes of neovascular glaucoma treatment are determined not only by the selected intervention but also by interactions among systemic inflammation, ocular microcirculation, and the structural stage of anterior segment involvement.

## Discussion

The present findings confirm that long-term response to treatment in neovascular glaucoma (NVG) is determined not only by the selected IOP-lowering strategy but primarily by the patient’s baseline systemic inflammatory and microcirculatory profile and the anatomical stage of iridocorneal angle involvement.

In our cohort, patients who underwent surgical intervention exhibited a less favorable systemic phenotype at baseline, characterized by higher SII, SIRI, and AISI indices, along with impaired ocular microcirculation reflected by a lower rheographic coefficient. These observations are consistent with the current understanding of NVG as a chronic ischemic-inflammatory disorder [3,18], characterized by high fibrogenic potential and pronounced structural remodeling of the iridocorneal angle during neovascularization [5,6,19].

This pathophysiological background may partly explain why, in real-world clinical practice, surgical procedures are more frequently applied in clinically complex patients and why direct comparisons of treatment efficacy without adjustment for angle stage and systemic status often yield inconsistent results [20-22]. Thus, treatment allocation itself reflects the disease’s biological severity and should be considered when interpreting clinical outcomes.

A key finding of this study was that the stage of iridocorneal angle neovascularization is an important determinant of treatment effectiveness. In the early stages, both strategies achieved high clinical success rates (surgery: 100%; cyclophotocoagulation: 92.0%). In contrast, at advanced stages (III-IV), surgical efficacy declined markedly to 33.3%, whereas cyclophotocoagulation maintained higher success rates (65.2%; p = 0.006).

Such a turning point is pathophysiologically justified, as active iris rubeosis, the formation of fibrovascular membranes, and complete angle obliteration create conditions for persistent elevation of intraocular pressure. In addition, postoperative inflammation—already markedly increased in these patients preoperatively—together with a pronounced tendency toward fibrosis and scarring, may compromise the long-term success of filtration surgery [3,5,6,19].

In contrast, cyclodestructive procedures are less dependent on the integrity of the trabecular outflow pathway, as they primarily reduce aqueous humor production. This mechanism provides a pathophysiological explanation for their relative stability in late-stage NVG [10,18]. Thus, the stage of iridocorneal angle neovascularization serves not only as a clinical marker of disease severity but also as a key determinant in selecting the optimal treatment strategy.

Despite comparable final IOP at 24 months, the larger IOP reduction with TSCPC likely reflects a stronger pressure-lowering effect in eyes starting from a more refractory functional state. In contrast, better visual outcomes after surgery may partly reflect baseline visual potential and stage-adapted case selection. This observation is consistent with long-term outcome data for both cyclophotocoagulation and filtration surgery [23-25].

Surgical interventions demonstrated significantly better functional visual outcomes (LogMAR 0.77 vs. 1.40; p = 0.002), likely reflecting a more stable restoration of anterior segment perfusion and a less direct impact on ciliary structures, as has also been reported in studies evaluating trabeculectomy and glaucoma drainage devices [24,26].

The rate of neovascular regression was high in both groups and did not differ significantly (64.1% after surgery vs. 70.8% after cyclophotocoagulation; p = 0.50), supporting the central role of ischemia control and IOP stabilization in suppressing angiogenesis irrespective of intervention type, consistent with current concepts of NVG pathogenesis [1,27].

These observations suggest that angiogenesis control in NVG is primarily driven by reduction of ischemic stimulus and stabilization of ocular hemodynamics rather than by the specific IOP-lowering mechanism itself [28,29].

Regarding safety, complication rates were higher after surgical interventions (30.8% vs. 14.6%; p = 0.07), reflecting greater procedural invasiveness and the risk of postoperative inflammatory or hypotonic reactions, as documented in large studies of filtration surgery and drainage devices [24,26].

Conversely, cyclophotocoagulation — particularly when performed using lower-energy or micropulse protocols — demonstrated a lower rate of severe complications while maintaining a substantial hypotensive effect [23,25], highlighting its clinical value in patients with high systemic risk or limited visual potential.

An important contribution of the present study is the demonstration of systemic inflammation and microcirculatory status as independent biological determinants of long-term treatment response. Surgical failure was associated with markedly elevated inflammatory indices, and multivariable analysis confirmed an independent negative effect of SII on clinical success. These findings conceptually align with evidence linking systemic and local inflammation to postoperative fibrosis, progression of peripheral anterior synechiae, and filtration failure in NVG [6,18].

Conversely, a higher rheographic coefficient independently predicted favorable outcomes, underscoring the pivotal role of ocular microcirculation in determining clinical prognosis.

Our results were consistent with our previous work demonstrating the importance of systemic inflammation, endothelial activation, and metabolic dysregulation in the clinical course and treatment response of NVG [30]. The previously reported impact of ICAM-1 and HbA1c on diabetic NVG prognosis, as well as the utility of multifactorial predictive models, were further supported in the present study, where combined clinical, inflammatory, and microcirculatory parameters determined long-term treatment success, reinforcing the concept of personalized NVG management.

Another clinically relevant signal was the role of cardiovascular comorbidity. Its presence was associated with a reduced likelihood of treatment success [4,20], and patients with surgical failure demonstrated a higher prevalence of cardiovascular disease. This may reflect both a pro-inflammatory, pro-ischemic systemic milieu with endothelial dysfunction promoting postoperative fibrosis and practical clinical limitations in selecting aggressive surgical strategies in high-risk patients [4].

Taken together, these findings support a stage-adapted and biologically informed therapeutic approach: filtration surgery appears most justified in early stages with preserved anatomical reserve, whereas cyclophotocoagulation represents a more controlled and stable option in advanced rubeosis, high systemic risk, or limited functional prognosis [4,31].

Study limitations. This study had several limitations. First, its retrospective and multicenter design may have introduced heterogeneity in treatment strategies and postoperative management across participating centers. Second, treatment allocation was not randomized but reflected real-world clinical practice, which increases the risk of selection bias and potential confounding. Third, the relatively small number of patients within certain subgroups limits the statistical power of subgroup analyses and may affect the robustness of the findings. Therefore, further prospective studies and external validation in independent cohorts are warranted to confirm the stability and generalizability of the proposed model.

## Conclusion

The long-term effectiveness of treatment for secondary neovascular glaucoma is determined not only by the type of intervention but also by underlying pathophysiological factors, including the stage of iridocorneal angle neovascularization, the level of systemic inflammation, and the characteristics of ocular microcirculation. Advanced angle obliteration reduced the likelihood of treatment success nearly sixfold (OR = 0.18; p = 0.007).

Surgical intervention increased the odds of treatment success more than fivefold (OR = 5.4; p = 0.003); however, this advantage was predominantly observed in early-stage disease with preserved anatomical and functional reserve.

Systemic inflammation and microcirculatory parameters demonstrated independent prognostic value: elevated SII decreased the probability of a successful outcome (OR = 0.76; p = 0.009), whereas a higher rheographic coefficient was associated with improved prognosis (OR = 1.42; p = 0.023), supporting the need for a personalized treatment selection strategy in neovascular glaucoma.
